# Rotate: A command-line program to rotate circular DNA sequences to start at a given position or string

**DOI:** 10.12688/wellcomeopenres.19568.1

**Published:** 2023-09-13

**Authors:** Richard Durbin, Bianca De Sanctis, Moritz Blumer

**Affiliations:** 1 Department of Genetics, University of Cambridge, Cambridge, England, CB2 3EH, UK

**Keywords:** Genetics, mitochondrial DNA, plastid DNA, circular DNA, bioinformatics

## Abstract

Sequences derived from circular DNA molecules (i.e. most bacterial, viral and plastid genomes) are expected to be linearised and rotated to a common start position for most downstream analyses including alignment. Despite this being a common and straightforward task, available software is either limited to a small number of input sequences, lacks the option to specify a custom anchor string, or requires a commercial license. Here, we present rotate, a simple, open source command line program written in C with no external dependencies, which can rotate a set of input sequences to a custom anchor string (allowing for a specified number of mismatches), or offset the input sequences to the desired position. The combination of both functionalities allows the rotation of all input sequences to any desired starting position, enabling downstream analysis. rotate is extremely fast and scales linearly with the number of input sequences, taking only seconds to rotate over a thousand mitochondrial sequences.

## Introduction

Some DNA sequences, such as most viral, bacterial and organelle genomes, are circular as opposed to linear. When these sequences are deposited into public repositories, they are formatted as linear sequences by assigning them starting positions and orientations. These starting positions and orientations can be somewhat arbitrary and often differ across individuals or taxa. However, most multiple sequence alignment programs (e.g. MAFFT ^1^, MUSCLE ^2^) assume linearity of sequences, including the same starting position. Performing accurate downstream analyses, then, requires first standardizing both their orientation and starting position.

Multiple programs or software packages already exist with some sequence rotation functionality, but have various restrictions on input, extensive dependencies, or do not allow user-defined starting positions or anchor strings. For example, geneious ^3^ can offset a sequence to a user-defined starting position, but does not allow automated rotation to a custom anchor string and it requires a commercial license. CSA ^4^ is restricted to 32 total input sequences, and rotates to an "optimal" rotation for multiple sequence alignment instead of a user-defined starting position. Circlator’s fixstart function ^5^ does not accept a user-defined starting position or anchor string, and instead tries to detect dnaA genes to guide the rotation, and the software has many dependencies (including BWA ^6^, Prodigal ^7^, MUMmer ^8^, and Canu ^9^ or SPAdes ^10^). Similarly to CSA, MARS ^11^ uses a sophisticated algorithm to compute the optimal rotation and can even integrate this into a multiple alignment algorithm, but again does not allow for a user-specified string or position. Though an "optimal" rotation is desirable in many contexts, the ability to rotate to a user-defined sequence or position is highly valuable, for example because it allows for the iterative inclusion of new sequences without re-running the algorithm on the entire dataset. Here, we present a software tool which can rotate a set of input sequences to a custom anchor string (allowing for a specified number of mismatches), or offset the input sequences to the desired position.

## Methods

### Implementation

rotate is a command-line program written in C that takes as input an (optionally gzipped) FASTA file of DNA sequences and either a new starting position (offset in base pairs) or anchor string which defines a new starting position, and outputs a FASTA file with the same sequences appropriately rotated. If a string is given as input, it can allow for any number of mismatches up to a user-defined threshold, and will also search for and output reverse complements when necessary. The program will fail if the specified input string is not unique in a target sequence, while allowing for mismatches, and return the locations instead. It is available at https://github.com/richarddurbin/rotate (see *Software availability* for more information).

### Operation

rotate has no external dependencies and is called from the command line. It accepts several arguments to invoke the desired functions, which are explained at https://github.com/richarddurbin/rotate. rotate is extremely fast – its runtime scales linearly with the number of input sequences, since every sequence is processed separately. rotate is easy to compile, and we tested its functionality on macOS 12.5.1 and on Scientific Linux 7.9.

## Use cases

Below we give an example of how to use rotate in combination with a multiple sequence alignment program of choice to rotate a large dataset of sequences to a common start position. To enhance reproducibility, we made the input data and an extended version of this use case available at https://github.com/MoritzBlumer/rotate_use_case. Briefly, we downloaded two sets of publicly available organelle assemblies from NCBI’s RefSeq database: (1) all available complete mammalian mitochondrial genomes (n=1,546) and (2) all available complete chloroplast genomes of the Rosaceae family (n=465) (as of 24 January 2023). For the mitochondria, we selected a conserved mammalian mitochondrial sequence (using the 100 Vertebrate Cons track in the UCSC Genome Browser ^12^) as the anchor string, while for the chloroplasts we used a common barcode primer ^13^. See *Underlying data* for more information. We first rotated the sequences with rotate, specifying the anchor string (-s) and maximum number of mismatches (-m), respectively. Execution of this first step on a Linux machine using a single CPU with 6GB RAM took 3.165 and 3.422 seconds to rotate 1,546 mitochondrial and 465 chloroplast sequences, respectively (wall clock time). Next, we generated a multiple sequence alignment for both rotated sets of sequences with MAFFT (version 7.520) ^1^ and then performed a second rotation by position (-x) to accomplish conventional mitochondrial and chloroplast start positions in the multiple sequence alignments.


        ## 1,546 mitochondria

# rotate to anchor string, allowing for 4 mismatches
./rotate -s TACGACCTCGATGTTGGATCA -m 4 mammalia.fa > mammalia.rotated.fa

# align with mafft
./mafft mammalia.rotated.fa > mammalia.aligned.fa

# offset to common mitochondrial start position
./rotate -x 23859 mammalia.aligned.fa > mammalia.final.fa

## 465 chloroplasts

# rotate to anchor string, allowing for 1 mismatch
./rotate -s CGAAATCGGTAGACGCTACG -m 1 rosaceae.fa > rosaceae.rotated.fa

# align with mafft
./mafft rosaceae.rotated.fa > rosaceae.aligned.fa

# offset to conventional chloroplast start position
./rotate -x 163249 rosaceae.aligned.fa > rosaceae.final.fa
      

**Figure 1.**
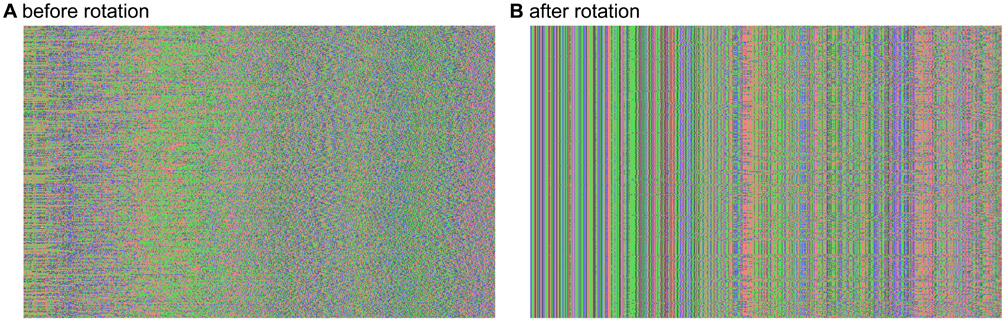
Visualization of the first 750 base pairs of 465 chloroplast assemblies ( **A**) before rotation and ( **B**) after rotation to a shared anchor sequence. No multiple sequence alignment was performed here. The sequence files were visualized using Aliview version 1.27 ^14^.

For illustration, the first 750 base pairs of the 465 chloroplast sequences are shown in Figure 1, with the raw sequences from rosaceae.fa in panel (A) and the rotated sequences from rosaceae.rotated.fa in panel (B). The sequence files were visualized using Aliview version 1.27 ^14^. Note that neither panel in Figure 1 has undergone multiple sequence alignment.

## Conclusions

Here we have presented a fast and simple command-line program to rotate circular DNA sequences, for example chloroplast or mitochondrial sequences, to a common starting position. This is often required to create a multiple sequence alignment. rotate can account for an arbitrary number of mismatches, has no external dependencies, and can process thousands of sequences in seconds.

## Data Availability

The two sets of publicly available organelle assemblies used in the use cases section of this study were sourced from from NCBI’s RefSeq database (on 24 January 2023), which comprised: All available complete mammalian mitochondrial genomes (n=1,546). We selected a conserved mammalian mitochondrial sequence (TACGACCTCGATGTTGGATCA) (using the 100 Vertebrate Cons track in the UCSC Genome Browser ^12^) as the anchor string. All available complete chloroplast genomes of the Rosaceae family (n=465). We used a common barcode primer (CGAAATCGGTAGACGCTACG) ^13^. For the complete use case including the used input data, see https://github.com/MoritzBlumer/rotate_use_case

## Software availability

Source code avaialble from: https://github.com/richarddurbin/rotate

Archived source code at time of publication: https://doi.org/10.5281/zenodo.8127980 ^15^

License: MIT
