# Supplementary material for: Rotate: A command-line program to rotate circular DNA sequences to start at a given position or string
Source: Wellcome Open Res. 2023 Sep 13;8:401. [Version 1] doi: 10.12688/wellcomeopenres.19568.1 (PMC11056001; doi:10.12688/wellcomeopenres.19568.1)
Supplement: Peer review history

# Reviewer report: Reviewer response for version 1

**DOI:** 10.21956/wellcomeopenres.21678.r79586

**Published:** 2024-05-08

**Authors:** João Gabriel Rodinho Nunes Ferreira

**Affiliations:** 1 Universidade Federal do Rio de Janeiro, Rio de Janeiro, State of Rio de Janeiro, Brazil

**# General Assessment**

 In this article, the authors address a challenge in the field of bioinformatics: the rotation of circular DNA sequences such as those found in most viral, bacterial, and organelle genomes. Typically, these sequences are deposited into public repositories as linear sequences with assigned starting positions and orientations, which can vary arbitrarily and affect downstream sequence alignment processes. Most existing sequence alignment programs assume a uniform linearity and starting point, which often does not reflect the circular nature of these genomes. To overcome those limitations, the authors developed rotate, a command-line tool that standardizes the starting positions and orientations of circular DNA sequences for more accurate downstream alignment in genomic analyses. Unlike previous tools, Rotate offers enhanced flexibility and scalability, supporting arbitrary mismatches and efficiently handling thousands of sequences simultaneously without the need for commercial licenses or extensive software dependencies.

 By providing a mechanism to standardize the orientation and starting positions of circular DNA sequences, the tool facilitates more accurate multiple sequence alignments, essential for subsequent analyses and comparative studies across different taxa. Its ability to process thousands of sequences rapidly, without external dependencies and regardless of sequence complexity, positions it as a valuable resource in genomic research. Such capabilities not only save valuable research time but also improve the reproducibility of scientific results, thereby contributing to advancements in the fields of genetics and molecular biology.

**# Detailed Feedback**

**## Manuscript**

 "The program will fail if the specified input string is not unique in a target sequence,"

     Could you clarify the behavior described where the program will fail if the specified input string is not unique in a target sequence? In my testing, I introduced a duplicate manually, and the program completed successfully (returning a status of 0). However, as expected the duplicated sequence was not included in the final FASTA output.

 "We selected a conserved mammalian mitochondrial sequence (TACGACCTCGATGTTGGATCA) (using the 100 Vertebrate Cons track in the UCSC Genome Browser12) as the anchor string"

     A more detailed explanation of how the conserved mammalian mitochondrial sequence was selected could greatly assist users. Providing this information would enable users to better understand the criteria and process involved, potentially guiding them in choosing appropriate anchor strings for their own datasets.

 On 'Fig.1':

     It does not include the mammalian sequences, despite the detailed description of their processing in the Use Cases section. To maintain consistency, I suggest either removing the detailed processing description of the mammalian sequences from the Use Cases section or adding their plots to Figure 1 alongside the Rosaceae sequences. This adjustment would enhance the coherence and completeness of the visual data presented.

     The primary purpose of 'rotate' is to prepare files for downstream analyses, such as multiple alignments. However, Figure 1 only presents a comparison between rotated and raw FASTA files without showing any actual alignment. To better support the tool's intended use, it would be valuable to include a comparison of aligned rotated sequences alongside aligned raw sequences. This would more effectively demonstrate the tool's efficacy in facilitating downstream analytical processes.

 "sourced from from NCBI’s RefSeq database"

     The word "from" is mistakenly repeated. It should read "sourced from NCBI’s RefSeq database."

**## Software**

 It would be helpful to provide details about what the program outputs to STDERR, as it seems to include statistics for each processed sequence, yet I found no specific documentation explaining these statistics. Additionally, considering implementing a 'verbose mode' (perhaps with a -v option) or directing these statistics to a separate file like 'rotation_stats.txt' could improve usability. This change would prevent the terminal from becoming cluttered with statistics when processing multiple sequences, thereby enhancing clarity and focus on the main output.

 It appears that if a sequence lacks a matching -s, it is almost silently discarded from final output FASTA; the only indication is that its statistics in STDERR comprise a single line, whereas other sequences display two. To enhance user clarity, the program could more explicitly notify users of non-matching sequences, either through a warning message or by generating a file listing the IDs of discarded sequences.

Are the conclusions about the tool and its performance adequately supported by the findings presented in the article?

Yes

Is the rationale for developing the new software tool clearly explained?

Yes

Is the description of the software tool technically sound?

Yes

Are sufficient details of the code, methods and analysis (if applicable) provided to allow replication of the software development and its use by others?

Yes

Is sufficient information provided to allow interpretation of the expected output datasets and any results generated using the tool?

Yes

Reviewer Expertise:

genomics

I confirm that I have read this submission and believe that I have an appropriate level of expertise to confirm that it is of an acceptable scientific standard.

# Reviewer report: Reviewer response for version 1

**DOI:** 10.21956/wellcomeopenres.21678.r79585

**Published:** 2024-04-27

**Authors:** Tobias Jakobi

**Affiliations:** 1 College of Medicine Phoenix, The University of Arizona, Phoenix, Arizona, USA

In their manuscript, Durbin et al. present rotate, a command line tool designed to aid in the task of correctly rotating circular DNA sequences required align multiple sequences based on custom offsets or sequences motifs. The software is very streamlined, uses no external dependencies and should work on any system that has a working C compiler. It is small and fast enough to easily integrate into existing software pipelines, e.g. for submission of sequences to the Genbank.

 The manuscript is well written and clearly documents the software’s functionality as well as performed benchmarks and tests. Reasoning for why a new software is required are clearly laid out. The benchmark documentation is available as a second repository and contains all necessary steps to perform the analysis.

 Minor:

 1) The authors might think about also including a short paragraph describing the second utility in the GitHub repository, `compose`.

Are the conclusions about the tool and its performance adequately supported by the findings presented in the article?

Yes

Is the rationale for developing the new software tool clearly explained?

Yes

Is the description of the software tool technically sound?

Yes

Are sufficient details of the code, methods and analysis (if applicable) provided to allow replication of the software development and its use by others?

Yes

Is sufficient information provided to allow interpretation of the expected output datasets and any results generated using the tool?

Yes

Reviewer Expertise:

Bioinformatician working in the field of circular RNAs a field closely related to circular DNAs in terms of algorithms and software.

I confirm that I have read this submission and believe that I have an appropriate level of expertise to confirm that it is of an acceptable scientific standard.

# Reviewer report: Reviewer response for version 1

**DOI:** 10.21956/wellcomeopenres.21678.r75613

**Published:** 2024-04-22

**Authors:** Benjamin Lee

**Affiliations:** 1 University of Oxford, Oxford, England, UK; 2 National Center for Biotechnology Information, National Library of Medicine, National Institutes of Health, Bethesda, Maryland, USA

The Rotate tool is a useful utility for analyzing sequences where an expected substring is present.

 The introduction covers the state of the art in circular sequence rotation. An additional tool you may want to include in the introduction is SeqKit, which has a "restart" command in v2. Again, it does not allow a user specified string for the start.

 Using the provided install command (i.e., clone the repo and run make), I was able to build the tool on a Apple Silicon and macOS 14.3.1.

 For my use case, aligning viroids with an expected hammerhead ribozyme motif (CTGANGA), the ability to tolerate mismatches is very useful. However, I have run into a minor issue you may wish to consider. The command line usage states "use either -x and optionally -rc, or -s" Viroids may have their ribozyme on either polarity (or indeed both). It would appear that -rc and -s are incompatible but the command does still execute. However, when it finds a matching string in the opposite polarity, the output sequence is in the same polarity as the input sequence, not as the search substring. 

 For example, this command:

 ./rotate -m 1 -rc -s  CTGANGA ../research/viroiddb/db/2021-09-07/avsunviroidae.fasta

  Includes in its output this line:

 >AF404052.1

 TGTTCTTCCCATCTTTCCCTGAAGTGACGAAGTGATCAAGAGATTGAAGACGAGTGAACTAATTTTTTTTTAATAAAAAGTTCACCACGACTCCTCCTTCTCTCACAAGTCGAAACTCAGAGTCGGAAAGTCGGAACAGACCTGGTTTCGTCAAACAAAGTTTAATCATATCCTCACTTCTTGTTCTATTTAACAAGATTTTGTAAAAAAACAATGAAGATAGAGGAGTAAACCTCGCGAGACTCATCAG

 The reverse complement of the final seven is indeed the search string but the output would not be directly usable. I would suggest either actively disallowing -rc and -s to be used together or add the additional logic to output the reverse complement.

 Finally, the ability to use ambiguous nucleotides would be helpful. I saw some references to ambiguity in seqio.c but it is not clear if that code is used in Rotate. Attempting to use ambiguous nucleotides in the search did not appear to work unless -m was used but this does not seem as it it would be useful for anything other than N.

 Overall, a useful contribution to the community and a tool I expect I will be using myself in the future.

Are the conclusions about the tool and its performance adequately supported by the findings presented in the article?

Yes

Is the rationale for developing the new software tool clearly explained?

Yes

Is the description of the software tool technically sound?

Yes

Are sufficient details of the code, methods and analysis (if applicable) provided to allow replication of the software development and its use by others?

Yes

Is sufficient information provided to allow interpretation of the expected output datasets and any results generated using the tool?

Yes

Reviewer Expertise:

Computational biology

I confirm that I have read this submission and believe that I have an appropriate level of expertise to confirm that it is of an acceptable scientific standard.

## References

[1] : SeqKit2: A Swiss army knife for sequence and alignment processing. *iMeta* .2024; 10.1002/imt2.191 10.1002/imt2.191 PMC1118319338898985

[2] : SeqKit: A Cross-Platform and Ultrafast Toolkit for FASTA/Q File Manipulation. *PLoS One* .2016;11(10) : 10.1371/journal.pone.0163962 e0163962 10.1371/journal.pone.0163962 27706213 PMC5051824
